# Heterogeneity in homogeneous nucleation from billion-atom molecular dynamics simulation of solidification of pure metal

**DOI:** 10.1038/s41467-017-00017-5

**Published:** 2017-04-05

**Authors:** Yasushi Shibuta, Shinji Sakane, Eisuke Miyoshi, Shin Okita, Tomohiro Takaki, Munekazu Ohno

**Affiliations:** 1 Department of Materials Engineering, The University of Tokyo, 7-3-1 Hongo, Bunkyo-ku, Tokyo 113-8656 Japan; 2 Graduate School of Science and Technology, Kyoto Institute of Technology, Matsugasaki, Sakyo-ku, Kyoto 606-8585 Japan; 3 Faculty of Mechanical Engineering, Kyoto Institute of Technology, Matsugasaki, Sakyo-ku, Kyoto 606-8585 Japan; 4 Division of Materials Science and Engineering, Faculty of Engineering, Hokkaido University, Kita 13 Nishi 8, Kita-ku, Sapporo, Hokkaido 060-8628 Japan

## Abstract

Can completely homogeneous nucleation occur? Large scale molecular dynamics simulations performed on a graphics-processing-unit rich supercomputer can shed light on this long-standing issue. Here, a billion-atom molecular dynamics simulation of homogeneous nucleation from an undercooled iron melt reveals that some satellite-like small grains surrounding previously formed large grains exist in the middle of the nucleation process, which are not distributed uniformly. At the same time, grains with a twin boundary are formed by heterogeneous nucleation from the surface of the previously formed grains. The local heterogeneity in the distribution of grains is caused by the local accumulation of the icosahedral structure in the undercooled melt near the previously formed grains. This insight is mainly attributable to the multi-graphics processing unit parallel computation combined with the rapid progress in high-performance computational environments.

## Abstract (web summary)

Nucleation is a fundamental physical process, however it is a long-standing issue whether completely homogeneous nucleation can occur. Here the authors reveal, via a billion-atom molecular dynamics simulation, that local heterogeneity exists during homogeneous nucleation in an undercooled iron melt.

## Introduction

Nucleation is the origin of all pattern formation phenomena via a first-order phase transformation, such as the formation of solidification microstructures, bubbles, liquid droplets and so on^1^. Nucleation is classified into homogeneous and heterogeneous cases^1–3^. Heterogeneous nucleation always occurs at nucleation sites in the walls of a mould or in impurities. Therefore, the number and variety of nucleation sites for heterogeneous nucleation have been widely controlled to obtain the desired microstructure for many years^2, 3^. On the other hand, it is still challenging to clarify the details of homogeneous nucleation since it only occurs in ideal cases. For example, it is not straightforward to accurately predict the size distribution of nuclei or the average distance between nuclei. Moreover, it is still unclear how accurately classical nucleation theory captures the nature of nucleation. In fact, many non-classical nucleation theories have been reported^4, 5^. Indeed, can completely homogeneous nucleation occur? This is a critical question for a wide range of phenomena involving a first-order phase transformation.

Since it is not straightforward to observe homogeneous nucleation by an experimental approach, computational approaches have been utilised to clarify each stage of the process. In general, mesoscale simulations including the Monte Carlo model^6, 7^, the cellular automaton model^8, 9^ and the phase-field model^10–16^ have successfully shed light on the nature of microstructure evolution. However, it is generally impossible to treat nucleation inherently in these mesoscale models since they are based on phenomenological models. Therefore, to treat nucleation, nuclei are specified in advance or are formed during the simulation in accordance with various rules to obtain a reasonable microstructure. To overcome these problems, it is essential to have knowledge at the atomistic scale. Therefore, molecular dynamics (MD) simulations have been widely employed to clarify the nature of nucleation from an atomistic viewpoint. For example, the existence of transient clusters during nucleation^17–19^, estimation of the nucleation rate and the nucleation barrier^20–22^, non-classical behaviour in nucleation^4, 5^ and nucleation in nanoparticles^23, 24^ have been investigated in detail by MD simulation. Although these studies have successfully captured the local structure in the nucleation, most of them were limited to the formation of a single nucleus (or a few nuclei) owing to the computational limitation, except for several pioneering works^21, 25^.

Recently, graphics processing unit (GPU)-accelerated computing has been focused on as a powerful tool for numerical simulations^26^. The expansion of MD simulations up to ten million atoms has been easily achieved even with the use of a single GPU^27–33^, since a GPU consists of many cores. Moreover, we have successfully applied a parallel GPU computational scheme^34, 35^ to very large scale MD simulations of up to a billion atoms. By utilising this cutting-edge technique, here we investigate homogeneous nucleation from an undercooled iron melt using a large scale MD simulation. The billion-atom MD simulation enables investigation of homogeneous nucleation from a statistical viewpoint. In particular, here we reveal the local heterogeneity in homogeneous nucleation and discuss the difficulty of completely homogeneous nucleation from an atomistic viewpoint, which cannot be conceived from a macroscopic perspective. For consistency with previous studies^27–33^, the nucleation from the undercooled iron melt is examined using the Finnis−Sinclair (FS) potential^36^ (see Methods for more information).

## Results

### Visualisation of nucleation and solidification processes

**Fig. 1:**
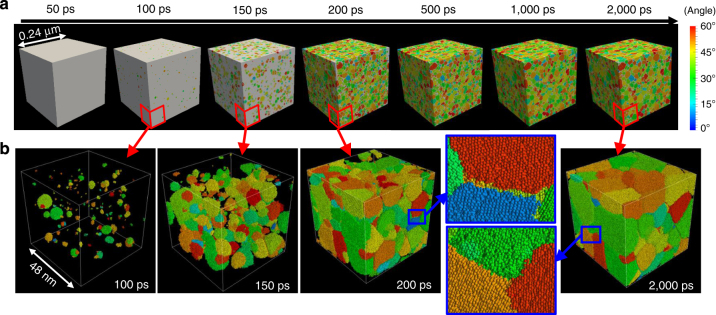
Snapshots of the simulation cell during a 2,000 ps calculation for a temperature of 0.58*T*_m_. **a** Whole simulation cell and **b** part of the simulation cell indicated by the *red lines* in **a**. All grains are coloured in accordance with the disorientation angle relative to the coordination axis. Areas of liquid and grain boundaries are coloured in *white* in **a** and are not shown in **b**. Inset figures represent enlarged views of the atomic configuration in the areas indicated by *blue rectangles*

Firstly, we discuss the nucleation from an undercooled iron melt and the subsequent microstructure formation at the critical undercooling temperature, where the finest microstructure should be obtained. It has been confirmed from previous MD studies using the FS potential that a nose peak exists in the nucleation rate with respect to temperature at around 0.58*T*_m_^30^ (see Supplementary Figure 1), where *T*_m_ represents the melting point of iron for the FS potential^37^ (see Methods for more information). Here, the same temperature of 0.58*T*_m_ is used. Figure 1a shows snapshots of the whole simulation cell during a 2000 ps calculation for the temperature of 0.58*T*_m_. All grains larger than 1.0 nm are coloured in accordance with the disorientation angle relative to the coordination axis (see Supplementary Note 2 for technical details), and the area of the liquid is coloured in white. Figure 1b shows snapshots of the atomic configuration in part of the simulation cell (with one-fifth of the length of the whole cell) at several time steps. All atoms belonging to grains larger than 0.25 nm are coloured in accordance with the disorientation angle relative to the coordination axis. The other atoms are not shown for clarity of the images. In this study, the term “grain” is used for solid assemblies in the simulation cell since it is not conceptually straightforward to distinguish nuclei and grains. Many small grains with a uniform distance between them appear in the simulation cell at 100 ps and grow larger with time. Further nucleation also occurs in the remaining part of the undercooled melt. By 200 ps, solidification is almost complete and therefore almost all the spaces are filled with many grains having various orientations, which form a fine microstructure. After that, the small grains shown in the snapshot for 200 ps at the atomistic scale (Fig. 1b) disappear by 2,000 ps (such as the red grains at the top boundary). Therefore, it is confirmed that grain coarsening occurs at this stage.

**Fig. 2:**
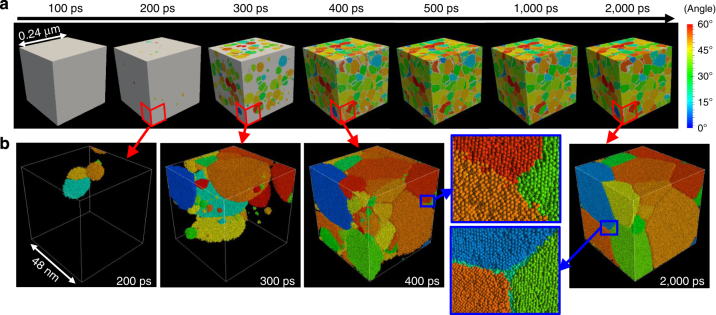
Snapshots of the simulation cell during a 2,000 ps calculation for a temperature of 0.67*T*_m_. **a** Whole simulation cell and **b** part of the simulation cell indicated by *red lines* in **a**. All grains are coloured in accordance with the disorientation angle relative to the coordination axis. Areas of liquid and grain boundaries are coloured in *white* in **a** and are not shown in **b**. Inset figures represent enlarged views of the atomic configuration in the areas indicated by *blue rectangles*

Next, the nucleation and microstructure formation at a higher temperature (i.e., a lower undercooling) of 0.67*T*_m_ are examined. Figure 2a and b, respectively, show snapshots of the whole simulation cell and the atomic configuration in part of the simulation cell during a 2,000 ps calculation for 0.67*T*_m_, which are drawn in the same manner as in Fig. 1. At this temperature, grains also appear at 200 ps. However, the number of grains is much smaller than that for 0.58*T*_m_. In the snapshot of the atomic configuration in part of the cell at 300 ps, the grains are not distributed uniformly but are concentrated around the grains already existing at 200 ps. In addition, small satellite-like grains exist near the previously formed large grains. Therefore, it is considered that the nucleation behaviour is different for the two cases with different temperatures. By 400 ps, the remainder of the melt has almost solidified, where small grains tend to exist. As in the case of 0.58*T*_m_, grain coarsening occurs and most of the small grains observed in the snapshots at 400 ps disappear by 2,000 ps. The obtained microstructure for 0.67*T*_m_ is coarser than that for 0.58*T*_m_.

### Transition in number of grains and grain size distribution

**Fig. 3:**
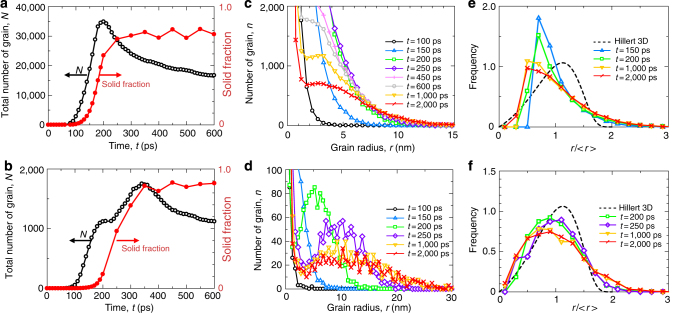
Transition in the number of grains and the grain size distribution. **a**, **b** Time changes of the total number of grains in the simulation cell and the solid fraction for **a** 0.58*T*_m_ and **b** 0.67*T*_m_. **c**, **d** Grain size distribution for several time steps for **c** 0.58*T*_m_ and **d** 0.67*T*_m_. **e**, **f** Grain size distribution normalised by the average grain radius for several time steps for **e** 0.58*T*_m_ and **f** 0.67*T*_m_

Figure 3a and b show the time changes of the total number of grains in the simulation cell and the solid fraction for 0.58*T*_m_ and 0.67*T*_m_, respectively. Here, the number of grains with a radius of 1.0 nm or more is counted. The solid fraction is defined as the ratio of atoms with the body-centred-cubic (bcc) configuration obtained from common neighbour analysis (CNA) to the total number of atoms (see Methods for more information). Note that the solid fraction does not reach one even when the solidification is complete since the atoms at the grain boundary are not classified as having the bcc configuration in CNA. In the case of 0.58*T*_m_, the total number of grains increases rapidly from approximately 100 ps after an incubation period. Then, it reaches a maximum of approximately 35,000 grains at around 200 ps, then gradually decreases. The solid fraction becomes saturated shortly after the number of grains reaches a maximum. On the other hand, two different rates of growth appear during the increase in the total number of grains for the case of 0.67*T*_m_. That is, the slope of the total number of grains with respect to time decreases after approximately 200 ps even though the total number of grains continues to increase until 350 ps. The solid fraction is still low at 200 ps, after which it rapidly increases, while the slope of the total number of grains with respect to time decreases. After 350 ps, the total number of grains gradually decreases.

Figure 3c and d show the grain size distribution at several time steps for 0.58*T*_m_ and 0.67*T*_m_, respectively. The grain size distribution decreases monotonically with increasing grain size at the initial stage for both temperatures, exhibiting a typical size distribution for steady-state nucleation^38^. In the case of 0.58*T*_m_, a shoulder appears in the grain size distribution after 450 ps, which means that larger grains grow at the expense of smaller grains in the system. Note that the solid fraction takes an almost constant value around 450 ps, while the total number of grains starts decreasing after 200 ps. Therefore, the solidification proceeds mainly by homogeneous nucleation before 200 ps and then by competitive growth between nuclei. Such typical nucleation and growth are also observed in the case of 0.67*T*_m_, although an important difference appears in this case: a shoulder appears in the grain size distribution at around 200 ps. Note that the total number of grains exhibits a plateau at around 200 ps in Fig. 3b, which indicates that homogeneous nucleation has almost ceased. Hence, at 0.67*T*_m_, typical nucleation and growth take place before 200 ps. However, the solid fraction continues to increase and, in particular, the total number of grains starts to increase again after 200 ps. This process is related to local heterogeneity, more specifically, the formation of satellite-like small grains near previously existing grains, which is discussed later.

Figure 3e and f show the grain size distribution normalised by the average grain radius for several time steps. Here, the number of grains with a radius of 2.0 nm or more is counted. In the case of 0.58*T*_m_, the distribution increases with decreasing normalised radius, then suddenly decreases owing to the use of a low-pass filter of 2.0 nm, whereas the distribution for 0.67*T*_m_ increases and decreases smoothly. Note that the data at the two early time periods represent the distribution of solid grains growing in the liquid, while the data at *t* = 1,000 and 2,000 ps correspond to the size distribution of grains in the polycrystalline system after the solidification at both temperatures. Although the grain size distribution at 0.67*T*_m_ basically resembles that from derived Hillert’s three-dimensional model^39^, it is broader than that derived from the ideal model. This is in agreement with most previous reports^13, 14^ since it is known that Hillert’s model predicts a narrower distribution owing to the use of mean-field theory.

### Distribution of disorientation angle for all grains

**Fig. 4:**
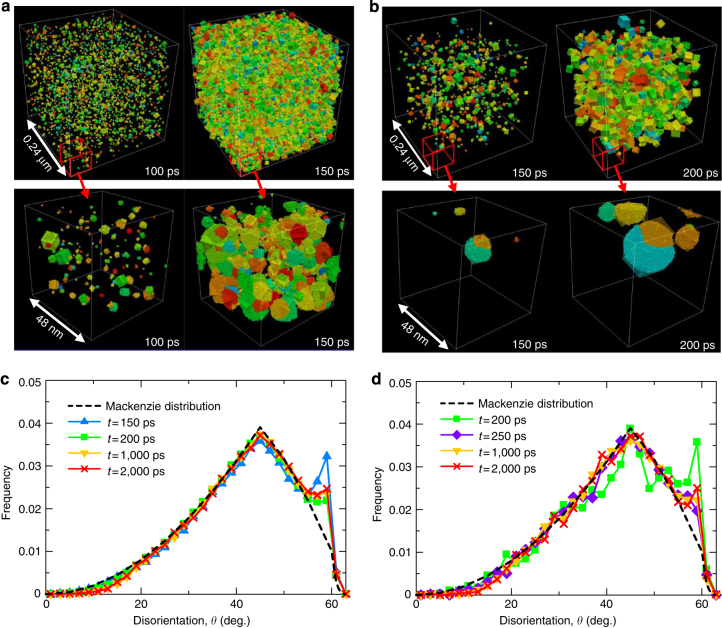
Distribution of disorientation angle for all grains. **a**, **b** Spatial distribution of disorientation angle of each grain relative to the coordination axis for **a** 0.58*T*_m_ and **b** 0.67*T*_m_. Cubes are located at the centres of grains. The volume and orientation of each cube are equivalent to the volume of the grain and the disorientation angle relative to the coordination axis, respectively. **c**, **d** Distributions of disorientation angle between neighbouring grains for **c** 0.58*T*_m_ and **d** 0.67*T*_m_. The *dashed line* is the Mackenzie distribution function, representing a random orientation

Next, the distribution of the grain orientation is investigated in detail. Figure 4a and b show the spatial distribution of the disorientation angle of each grain relative to the coordination axis at 100 and 150 ps for 0.58*T*_m_ and at 150 and 200 ps for 0.67*T*_m_, respectively. The superimposed cubes are located at the centres of the corresponding grains in the figures (see Methods for more information). The volume and orientation of each cube are equivalent to the volume of the grain and the disorientation angle relative to the coordination axis, respectively. Then, the disorientation angle between neighbouring grains is calculated for all neighbouring grains for several time steps. Figure 4c and d shows the distributions of the disorientation angle between neighbouring grains for 0.58*T*_m_ and 0.67*T*_m_, respectively. The *dashed line* is the Mackenzie distribution function^40^, which represents a random orientation. The distribution of the disorientation angle between neighbouring grains approximately follows the Mackenzie distribution, which means that the grains are basically randomly oriented in space. However, a clear peak is observed at around 60° for both temperatures. Therefore, there should be a specific interaction at a disorientation angle of 60°. It is known that grain boundaries with Σ3 misorientations have a disorientation angle of 60°^41^. Among the grain boundaries with Σ3 misorientations, a coherent twin boundary with an extremely small grain boundary energy exists (bcc(112)Σ3 plane for the < 110 > tilt axis with a tilt angle of 109.47°)^37^. The existence of a twin boundary in grains is investigated in detail from an atomistic viewpoint in the following section.

Another characteristic of the distribution of the disorientation angle between neighbouring grains is the decrease at small disorientation angles of less than 20°. In particular, the deviation from the Mackenzie distribution becomes noticeable over time. In general, the grain boundary energy at a low-angle grain boundary decreases with decreasing misorientation angle^37^, which is known as the Read−Shockley relation^42^. Therefore, a low-angle grain boundary tends to decrease its misorientation angle by rotation to decrease the grain boundary energy. Actually, a Monte Carlo simulation revealed that the ratio of low-angle grain boundaries decreases when grain rotation is dominant during the grain growth^43^. Therefore, it is considered that the grain rotation occurs at the latter stage of the microstructure evolution in our MD simulation.

### Evolution of the twin boundary in grains

**Fig. 5:**
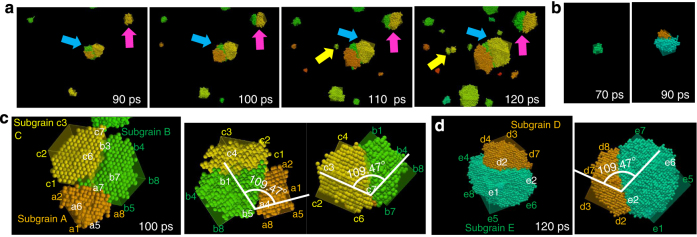
Snapshots of atomic configuration showing the evolution of twin boundaries in grains. **a**, **b** Time series of snapshots showing the evolution of twin boundaries for **a** 0.58*T*_m_ and **b** 0.67*T*_m_. **c**, **d** Enlarged views of grains with twin boundaries extracted from the calculation for **c** 0.58*T*_m_ and **d** 0.67*T*_m_. Vertices of cubes indicating the disorientation angle of the grain relative to the coordination axis are labelled to clarify the positional relationship

Figure 5a and b show snapshots of the atomic configuration, focusing on specific grains in the simulation cell, during the nucleation for 0.58*T*_m_ and 0.67*T*_m_, respectively. Grains consisting of subgrains with different orientations exist at both temperatures. It is confirmed from the snapshots that further nucleation occurs from the surface of a previously existing grain to form subgrain structures with different orientations (grains indicated by *magenta*, *yellow* and *blue arrows* in Fig. 5a, and the grain in Fig. 5b). It appears that heterogeneous nucleation occurs on the surface of the previously existing grain. It is known that planar defects (i.e., stacking faults) can form during the growth of a crystal from a melt and that defect formation does not require heterogeneous nucleation for the case of face-centred-cubic (fcc) metals^44^. This difference is discussed below from the viewpoint of the grain boundary energy. Figure 5c and d, respectively, show the detailed structures of the grain with the subgrain structure indicated by the *blue arrow* in Fig. 5a and the subgrain in Fig. 5b. The vertices of cubes indicating the disorientation angle of each grain relative to the coordination axis are labelled to clarify the positional relationships in the snapshots. The grain in Fig. 5c consists of subgrains A, B and C. It is confirmed that grain boundaries with a tilt angle of 109.47° exist along with a < 110 > tilt axis both between A and B and between B and C, which compose a coherent twin boundary. The grain in Fig. 5d consists of subgrains D and E. The grain boundary between subgrains D and E also has a tilt angle of 109.47° along with a < 110 > tilt axis.

Since a coherent twin boundary in bcc iron has an extremely small though non-zero grain boundary energy^37^, it is expected that grains with subgrains having a coherent twin boundary are easily formed during the solidification process. According to the Young’s relation^2^, the contact angle *θ* for the heterogeneous nucleation of a subgrain on the surface of a previously existing grain with a twin boundary is estimated as

1 $$\cos \,\theta =1-\frac{{\sigma }_{{\rm{GB}}}}{{\sigma }_{{\rm{SL}}}},$$

where *σ*_GB_ and *σ*_SL_ represent the grain boundary energy and the solid-liquid interfacial energy, respectively. When the typical values of *σ*_GB_ = 0.3 J m^−2^ for the twin boundary energy with a tilt angle of 109.47°^45^ and *σ*_SL_ = 0.2 J m^−2^ for the solid-liquid interfacial energy^2^ are employed, the contact angle is estimated to be 120°. The nucleation barrier for heterogeneous nucleation Δ*G*_hetero_ is defined as

2 $$\Delta {G}_{{\rm{hetero}}}=f(\theta )\Delta {G}_{{\rm{homo}}}=\frac{{(1-\cos \theta )}^{2}(2+\,\cos \,\theta )}{4}\Delta {G}_{{\rm{homo}}},$$

where Δ*G*_homo_ is the nucleation barrier for homogeneous nucleation^2^. For the contact angle of 120°, the factor *f*(*θ*) becomes 0.84, which indicates that this heterogeneous nucleation process is energetically preferable to the homogeneous nucleation process. On the other hand, the grain boundary energies for all other grain boundaries are at least 1.0 J m^−2^, in which heterogeneous nucleation does not occur since the right side of Eq. 1 becomes less than −1. Therefore, only the twin boundary with a tilt angle of 109.47° along with a < 110 > tilt axis can be formed via heterogeneous nucleation. On the other hand, planar defects (i.e., stacking faults) are easily formed in both nanoparticles^46^ and bulk metals^44, 47^ during the nucleation and solidification of fcc metals. It is known that the grain boundary energy of a twin boundary in fcc metals is almost zero, whereas that in bcc metals has a non-zero value^37^. From Eq. 1, the contact angle for heterogeneous nucleation is estimated to be zero when the grain boundary energy is zero. Therefore, it is natural that planar defects are easily formed in fcc metals in the MD simulation, whereas such stacking faults are not found in bcc iron according to our simulation. Therefore, heterogeneous nucleation is the dominant pathway to in the formation of twin boundaries in bcc metals during solidification, whereas it is possible to form planar defects without a nucleation event by chance owing to the thermal vibration at high temperatures. It is interesting to note that, from the MD simulation, coherent twin boundaries are formed via heterogeneous nucleation on the surface of previously existing grains despite the homogeneous nucleation elsewhere.

### Local heterogeneity in the spatial distribution of grains

**Fig. 6:**
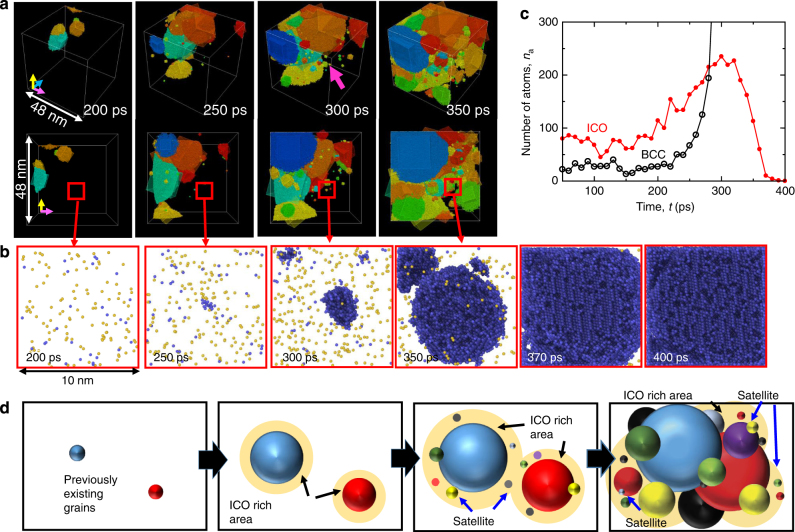
Satellite-like small grains near the surface of previously existing grains. **a** Snapshots of part of the simulation cell between 200 and 350 ps in the calculation for 0.67*T*_m_ (i.e., bird’s-eye and side views). **b** Snapshots of the atomic configuration in the part of the simulation cell (10 × 10 × 10 nm^3^) indicated by the *red squares* in **a**, where satellite-like small grains nucleate near one of the previously existing grains. Only atoms with the bcc (*blue atoms*) and ICO (*yellow atoms*) configurations, as classified by adaptive CNA, are shown for clarity of the figures. **c** Time changes in the numbers of atoms classified as having bcc and ICO configurations, where all the atoms in the cell in **b** are counted. **d** Schematic illustration of the formation process of satellite-like grains in ICO-rich areas near the surface of previously existing grains

Regarding the local heterogeneity, there is heterogeneity in the spatial distribution of the grains during the nucleation. Figure 6a shows snapshots of part of the simulation cell between 200 and 350 ps in the calculation for 0.67*T*_m_. As can be seen in the snapshots, grains are not distributed uniformly around 300 ps but accumulate around previously existing grains, as if they are satellites of such grains. Here, the local configuration of the undercooled melt where the satellite-like small grains appear is investigated in detail by adaptive CNA (see Methods for more information) to determine the physical origin of the local heterogeneity. Figure 6b shows snapshots of the atomic configuration in the part of the simulation cell (10 × 10 × 10 nm^3^) indicated by *red squares* in Fig. 6a, where a satellite-like small grain (indicated by the *magenta arrow* in Fig. 6a) nucleates near the previously existing large grains. In the snapshots, only atoms classified as having bcc and icosahedral (ICO) configurations by adaptive CNA are shown for clarity of the figures. The time changes in the numbers of atoms classified as having bcc and ICO configurations are shown in Fig. 6c, where all the atoms in the area in Fig. 6b are counted. Note that the existence of atoms with the ICO configuration in the undercooled melt implies Frank’s ICO short-range ordering of atoms in the liquid rather than stable quasicrystal phase. As shown in both the snapshots and the graph, the number of atoms with the ICO configuration increases after 200 ps, which is equivalent to the time when one of the previously existing large grains approaches the area in Fig. 6b. Therefore, it is expected that the local accumulation of atoms with the ICO configuration in the undercooled melt near the previously existing grains causes the nucleation of satellite-like small grains, which is discussed in detail in the following section.

There have been several studies focusing on the increase in the numbers of atoms with the ICO configuration in an undercooled melt prior to the nucleation of bcc and fcc phases in small systems^19, 31, 48, 49^. In particular, Kurtuldu and coworkers^48, 49^ confirmed by experimental observation that ICO quasicrystal-enhanced nucleation occurs in an iridium-added gold alloy. They proposed a model in which the local Frank’s ICO structure in the liquid is a template of the nucleation. The validity of their model was proved by examination of the orientation relationship between neighbouring grains, which indicated the presence of multiple twins with fivefold symmetry. Interestingly, Hou and coworkers^47, 50^ confirmed that multiple twinned grains with fivefold symmetry are formed after the accumulation of ICO atoms in MD simulations of the rapid quenching of liquid aluminium (i.e., an fcc metal). On the other hand, multiple twinned grains with fivefold symmetry were not formed in our simulation. As discussed above, the twin boundary energy for bcc iron is non-zero, which makes it difficult to induce the formation of twins with fivefold symmetry. However, multiple twinned grains were formed in our simulation as shown in Fig. 5c. Moreover, it is significant that the experimental observation of the disorientation angle distribution between neighbouring grains has a single peak at 60° whereas it basically agrees with the Mackenzie distribution, in agreement with our result. Since the experiment proved that the local Frank’s ICO structure in the liquid can be a template for nucleation, it is expected that the ICO template is the key for nucleation from a highly undercooled liquid melt even in our MD simulation. Therefore, it is considered that the local accumulation of atoms with the ICO configuration in the undercooled melt near the previously existing grains causes the nucleation of satellite-like small grains, as schematically illustrated in Fig. 6d. This discovery of the local inhomogeneity during homogeneous nucleation from the atomistic viewpoint is attributable to the very large scale MD simulation with a billion atoms.

## Discussion

**Fig. 7:**
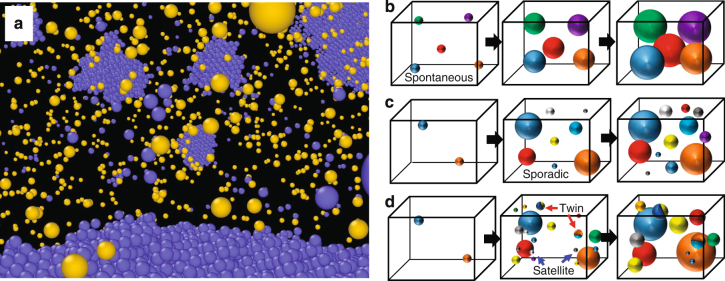
Local heterogeneity in spatial distribution of grains during homogeneous nucleation. **a** Snapshot of atomic configuration around the surface of a previously existing large grain at 300 ps for the 0.67*T*_m_ calculation. Only atoms with body-centred cubic (*blue atoms*) and ICO (*yellow atoms*) configurations, as classified by adaptive CNA, are shown. **b**-**d** Schematic illustration of models for homogeneous nucleation and solidification: **b** spontaneous nucleation model^51^, **c** sporadic nucleation model^51^ and **d** local heterogeneity model proposed in this study

As previously discussed, many satellite-like grains exist near the surface of the previously formed grains, which causes local heterogeneity in the spatial distribution of the grains during the homogeneous nucleation (Fig. 7a). Here, we propose a model for nucleation from the undercooled melt by comparison with representative classical models. There are two main classical models for isothermal crystallisation: spontaneous nucleation (Fig. 7b) and sporadic nucleation (Fig. 7c)^51^. That is, all growth starts at the same time in spontaneous nucleation, whereas the growth occurs at a constant rate in sporadic nucleation. These models do not take into account the specific distribution of grains but assume their uniform distribution. However, inhomogeneity appears in the spatial distribution of grains obtained from the large-scale MD simulation. Moreover, grains with a twin boundary are occasionally formed by heterogeneous nucleation at the surface of a previously existing grain. However, most of the satellite-like grains have random orientations that are independent of those of neighbouring previously existing grains. On the basis of these results, we propose a local heterogeneity model as a possible pathway for homogeneous nucleation from the undercooled melt of a pure metal as illustrated in Fig. 7d. That is, after an initial incubation, sporadic nucleation occurs from the undercooled melt at an almost constant rate at the initial stage since the number of grains increases almost linearly at this stage (Fig. 3b). Then, satellite-like grains nucleate near the surface of previously existing large grains. At the same time, grains with a twin boundary are formed by heterogeneous nucleation from the surface of the previously existing grains. The satellite-like grains become prominent as the temperature increases (i.e., away from the nose temperature), whereas twin boundaries appear at both temperatures (i.e., 0.58*T*_m_ and 0.67*T*_m_). Grain coarsening locally occurs in the area with close-packed grains before the solidification is complete. However, the microstructure obtained via this process has an almost random crystal orientation since most of the satellite-like grains have random orientations independent of those of neighbouring previously existing grains.

In summary, homogeneous nucleation from an undercooled iron melt and the subsequent solidification process were investigated by performing a large-scale MD simulation with a billion atoms on a GPU-rich supercomputer. The distribution of the disorientation angle basically satisfies the Mackenzie distribution, which means that the microstructure obtained via nucleation has a random crystal orientation, demonstrating the statistical validity of the large-scale simulation with a billion atoms. Moreover, the discovery of the spontaneous occurrence of local heterogeneity during homogeneous nucleation without any assumptions is a significant finding. Since some assumptions are required to obtain a predefined result in phenomenological methods including Monte Carlo and phase-field simulations, a major advantage of MD simulations is that they can reveal unknown phenomena without any predefined parameters. In particular, to demonstrate the local heterogeneity in the spatial distribution of grains by a large-scale simulation, a billion atoms were required since a large space is necessary for the local heterogeneity to emerge in the MD simulation. Such progress is mainly attributable to the multi-GPU parallel computation technique combined with the rapid progress in high-performance computational environments. The simulation cell had a side length of approximately 0.24 μm, making this the largest MD simulation of consecutive nucleation, solidification and grain growth in a metallic system ever to be reported to the best of our knowledge, although there have been several previous reports of large-scale MD simulations with more than a billion atoms for an ideal Lennard−Jones system^52, 53^.

## Methods

### MD simulation

The FS potential^36^ is employed for the interatomic potential between iron atoms, which is one of the most established potentials for bcc metals (see Supplementary Note 1 for more information). It has been confirmed in previous studies that the FS potential can reproduce various properties of bcc metals including nucleation from an undercooled iron melt^27–33^. A leapfrog method is used to integrate the classical equation of motion with a time step of 5.0 fs (energy conservation has been proven in previous constant-NVE simulations). A Berendsen thermostat^54^ is applied to control the temperature in each step, and the Andersen method^55^ is applied to independently control the pressure in each direction. The initial configuration of the calculation system is prepared by heating a bcc crystal of iron of size 236.8 × 236.8 × 236.8 nm (800 × 800 × 800 unit cells, 1,024,000,000 atoms) at 3000 K with a constant-NVT ensemble for 10 ps, which is followed by further relaxation at 2000 K for 200 ps and 1800 K for 200 ps. The prepared initial configuration is annealed isothermally in the main calculation for 2000 ps under zero pressure with the constant-NPT ensemble at 1600 or 1400 K. Since the melting point of bcc iron given by the FS potential is *T*_m_ = 2400 K^37^, which is higher than the experimental value of 1811 K, temperatures normalised by *T*_m_ are employed. That is, 1600 and 1400 K correspond to 0.67*T*_m_ and 0.58*T*_m_, respectively.

### Multi-GPU parallelization on GPU supercomputer

We have developed a parallel GPU code for multiple GPUs to accelerate the large-scale MD simulations. The code is written using the compute unified device architecture based on the C/C +  + language, and the message passing interface is used for internode communication. Supplementary Figure 2 shows a schematic illustration of the multi-GPU parallelization involving domain decomposition and data exchange between subdomains. The whole simulation cell is divided into subdomains and each subdomain is assigned to one GPU. The domain decomposition method^27, 56^ is also applied to accelerate the search for neighbouring atoms in each subdomain. Data exchange between the GPUs is performed for the data on the subdomain boundaries via host CPUs since a GPU cannot directly access the global memory of the other GPUs. In this study, the whole domain is divided into 512 (1 × 16 × 32) subdomains, and therefore 512 GPUs are parallelized for the large-scale MD simulation. All computations are performed on the GPU-rich supercomputer TSUBAME2.5, which has 1,408 nodes. One node consists of two CPUs (Intel, Xeon X5670) and three GPUs (NVIDIA, Tesla K20X).

### Post-analyses and visualisation

The obtained atomic configuration is first analysed by CNA to identify solid and liquid structures, basically following our previous study^24, 30^. The atoms with the bcc configuration are determined by considering the coordination numbers of the first-nearest-neighbour and second-nearest-neighbour atoms using two cutoff lengths. That is, atoms satisfying the following two conditions are identified as having the bcc configuration: (i) 8 neighbour atoms within a cutoff length of 2.75 Å, which is between the first-nearest-neighbour and second-nearest-neighbour atoms of the bcc crystal and (ii) 14 neighbour atoms within a cutoff length of 3.4 Å, which is between the second-nearest-neighbour and third-nearest-neighbour atoms of the bcc crystal. Next, the disorientation angle relative to the coordination axis is estimated for all atoms with the bcc configuration (see Supplementary Figure 3 and Supplementary Note 2 for details of the process). Then, the neighbouring atoms with a difference in the crystal orientation of less than 3° are classified as belonging to the same grain. The average disorientation angle for all atoms in the grain is regarded as the disorientation angle of the grain. All atoms are coloured in accordance with on the disorientation angle of the grain in the range from 0° to 62.80°^40^. Furthermore, the adaptive CNA method^57^, which employs variable cutoff distances, is used to precisely identify the ICO configuration in the undercooled melt. The adaptive CNA is performed using Open Visualisation Tool^58^.

### Data availability

The data that support the findings of this study are available from the corresponding author upon request.

## Electronic supplementary material

Supplementary Information Supplementary Figures, Supplementary Notes, Supplementary Table and Supplementary References

## References

[1] Onuki, A. *Phase Transformation Dynamics* (Cambridge University Press, 2002).

[2] Kurz, W. & Fisher, D. J. *Fundamentals of Solidification* (Trans Tech Publications, 1998).

[3] Dantzig, J. A. & Rappaz, M. *Solidification* (EPFL Press, 2009).

[4] Cherne FJ, Baskes MI, Schwarz RB, Srinivasan SG, Klein W (2004). Non-classical nucleation in supercooled nickel. Model. Simul. Mater. Sci. Eng..

[5] Russo J, Tanaka H (2016). Nonclassical pathways of crystallization in colloidal systems. MRS Bull..

[6] Anderson MP, Srolovitz DJ, Grest GS, Sahni PS (1984). Computer simulation of grain growth – I. Kinetics. Acta. Metall.

[7] Grest GS, Srolovitz DJ, Anderson MP (1985). Computer simulation of grain growth – IV. anisotropic grain boundary energy. Acta. Metall.

[8] Rappaz M, Gandin CA (1993). Probabilistic modelling of microstructure formation in solidification processes. Acta. Metall. Mater.

[9] Gandin CA, Rappaz M (1994). A coupled finite element-cellular automaton model for the prediction of dendritic grain structures in solidification processes. Acta. Metall. Mater.

[10] Steinbach I (1996). A phase field concept for multiphase systems. Physica D..

[11] Fan D, Chen L-Q (1997). Computer simulation of grain growth using a continuum field model. Acta. Mater..

[12] Chen L-Q (2002). Phase-field models for microstructure evolution. Annu. Rev. Mater. Res..

[13] Kim SG, Kim DI, Kim WT, Park YB (2006). Computer simulations of two-dimensional and three-dimensional ideal grain growth. Phys. Rev. E.

[14] Suwa Y, Saito Y, Onodera H (2007). Three-dimensional phase field simulation of the effect of anisotropy in grain-boundary mobility on growth kinetics and morphology of grain structure. Comput. Mater. Sci..

[15] Miyoshi E, Takaki T (2016). Validation of a novel higher-order multi-phase-field model for grain-growth simulations using anisotropic grain-boundary properties. Comput. Mater. Sci..

[16] Miyoshi E, Takaki T (2016). Extended higher-order multi-phase-field model for three-dimensional anisotropic-grain-growth simulations. Comput. Mater. Sci..

[17] Doliwa B, Heuer A (1998). Cage effect, local anisotropies, and dynamic heterogeneities at the glass transition: a computer study of hard spheres. Phys. Rev. Lett..

[18] Kob W (1999). Computer simulations of supercooled liquids and glasses. J. Phys. Condens. Matter.

[19] Hou ZY, Liu LX, Liu RS, Tian ZA, Wang JG (2010). Kinetic details of nucleation in supercooled liquid Na: a simulation tracing study. Chem. Phys. Lett..

[20] Auer S, Frenkel D (2001). Prediction of absolute crystal-nucleation rate in hard-sphere colloids. Nature.

[21] Aga RS, Morris JR, Hoyt JJ, Mendelev M (2006). Quantitative parameter-free prediction of simulated crystal-nucleation times. Phys. Rev. Lett..

[22] Bokeloh J, Rozas RE, Horbach J, Wilde G (2011). Nucleation barriers for the liquid-to-crystal transition in Ni: experiment and simulation. Phys. Rev. Lett..

[23] Shibuta Y, Suzuki T (2007). Melting and nucleation of iron nanoparticles: a molecular dynamics study. Chem. Phys. Lett..

[24] Shibuta Y, Suzuki T (2008). A molecular dynamics study of the phase transition in bcc metal nanoparticles. J. Chem. Phys..

[25] Streitz FH, Glosli JN, Patel MV (2006). Beyond finite-size scaling in solidification simulations. Phys. Rev. Lett..

[26] Shibuta Y, Ohno M, Takaki T (2015). Solidification in a supercomputer: from crystal nuclei to dendrite assemblages. JOM.

[27] Oguchi K, Shibuta Y, Suzuki T (2012). Accelerating molecular dynamics simulation performed on GPU. J. Jpn. Inst. Met.

[28] Shibuta Y, Oguchi K, Suzuki T (2012). Large-scale molecular dynamics study on evolution of grain boundary groove of iron. ISIJ. Int..

[29] Shibuta Y, Oguchi K, Ohno M (2014). Million-atom molecular dynamics simulation on spontaneous evolution of anisotropy in solid nucleus during solidification of iron. Scr. Mater.

[30] Shibuta Y, Oguchi K, Takaki T, Ohno M (2015). Homogeneous nucleation and microstructure evolution in million-atom molecular dynamics simulation. Sci. Rep.

[31] Shibuta Y, Sakane S, Takaki T, Ohno M (2016). Submicrometer-scale molecular dynamics simulation of nucleation and solidification from undercooled melt: linkage between empirical interpretation and atomistic nature. Acta. Mater..

[32] Okita S, Shibuta Y (2016). Grain growth in large-scale molecular dynamics simulation: linkage between atomic configuration and von Neumann-Mullins relation. ISIJ Int..

[33] Okita, S. et al. Molecular dynamics simulations investigating consecutive nucleation, solidification and grain growth in a twelve-million-atom Fe-system. *J. Cryst. Growth* doi: 10.1016/j.jcrysgro.2016.11.120 (2017).

[34] Shimokawabe, T. *et al.* Peta-scale phase-field simulation for dendritic solidification on the TSUBAME 2.0 supercomputer. In *Proceedings of 2011 International Conference for High Performance Computing, Networking, Storage and Analysis (SC)*, 1–11 (2011).

[35] Takaki T, Shimokawabe T, Ohno M, Yamanaka A, Aoki T (2013). Unexpected selection of growing dendrites by very-large-scale phase-field simulation. J. Cryst. Growth..

[36] Finnis MW, Sinclair JE (1984). A simple empirical N-body potential for transition metals. Philos. Mag. A.

[37] Shibuta Y, Takamoto S, Suzuki T (2008). A molecular dynamics study of the energy and structure of the symmetric tilt boundary of iron. ISIJ Int..

[38] Balluffi, R. W., Allen, S. M. & Carter, W. C. *Kinetics of Materials* (John Wiley & Sons, Inc., 2005).

[39] Hillert M (1965). On the theory of normal and abnormal grain growth. Acta Metall.

[40] Mackenzie JK (1958). Second paper on statistics associated with the random disorientation of cubes. Biometrika.

[41] Olmsted DL, Foiles SM, Holm EA (2009). Survey of computed grain boundary properties in face-centered cubic metals: I. Grain boundary energy. Acta. Mater..

[42] Read WT, Shockley W (1950). Dislocation models of crystal grain boundaries. Phys. Rev.

[43] Moldovan D, Wolf D, Phillpot SR, Haslam AJ (2002). Role of grain rotation during grain growth in a columnar microstructure by mesoscale simulation. Acta. Mater..

[44] Burke E, Broughton JQ, Gilmer GH (1988). Crystallization of fcc (111) and (100) crystal-melt interfaces: a comparison by molecular dynamics for the Lennard-Jones system. J. Chem. Phys..

[45] Wolf D (1990). Correlation between the energy and structure of grain boundaries in b.c.c. metals. II. Symmetrical tilt boundaries. Philos. Mag. A.

[46] Maekawa Y, Shibuta Y (2016). Dewetting dynamics of nickel thin film on alpha-quartz substrate: a molecular dynamics study. Chem. Phys. Lett..

[47] Hou ZY (2016). Cooling rate dependence of solidification for liquid aluminum: a large-scale molecular dynamics simulation study. Phys. Chem. Chem. Phys..

[48] Kurtuldu G, Sicco A, Rappaz M (2014). Icosahedral quasicrystal-enhanced nucleation of the fcc phase in liquid gold alloys. Acta. Mater..

[49] Rappaz M, Kurtuldu G (2016). Quasicrystal-enhanced nucleation during the solidification of fcc metallic alloys: a tentative thermodynamic approach. J. Phase Equilib. Diffus..

[50] Hou Z, Tian Z, Liu R, Dong K, Yu A (2015). Formation mechanism of bulk nanocrystalline aluminium with multiply twinned grains by liquid quenching: a molecular dynamics simulation study. Comput. Mater. Sci..

[51] Ibeh, C. C. *Thermoplastic Materials: Properties, Manufacturing Methods, and Applications* (CRC Press, 2011).

[52] Diemand J, Angélil R, Tanaka KK, Tanaka H (2013). Large scale molecular dynamics simulations of homogeneous nucleation. J. Chem. Phys..

[53] Watanabe H, Suzuki M, Ito N (2013). Huge-scale molecular dynamics simulation of multibubble nuclei. Comput. Phys. Commun..

[54] Berendsen HJC, Postma JPM, van Gunsteren WF, DiNola A, Haak JR (1984). Molecular dynamics with coupling to an external bath. J. Chem. Phys..

[55] Andersen HC (1980). Molecular dynamics simulations at constant pressure and/or temperature. J. Chem. Phys..

[56] Allen, M. P. & Tildesley, D. J. *Computer Simulation of Liquids* (Oxford Science Publications, 1987).

[57] Stukowski A (2012). Structure identification methods for atomistic simulations of crystalline materials. Model. Simul. Mater. Sci. Eng..

[58] Stukowski A (2010). Visualization and analysis of atomistic simulation data with OVITO–the open visualization tool. Model. Simul. Mater. Sci. Eng..

