# Supplementary material for: Heterogeneity in homogeneous nucleation from billion-atom molecular dynamics simulation of solidification of pure metal
Source: Nat Commun. 2017 Apr 5;8:10. doi: 10.1038/s41467-017-00017-5 (PMC5431899; doi:10.1038/s41467-017-00017-5)
Supplement: Supplementary file 1 — Supplementary Figures, Supplementary Notes, Supplementary Table and Supplementary References [file 41467_2017_17_MOESM1_ESM.pdf]

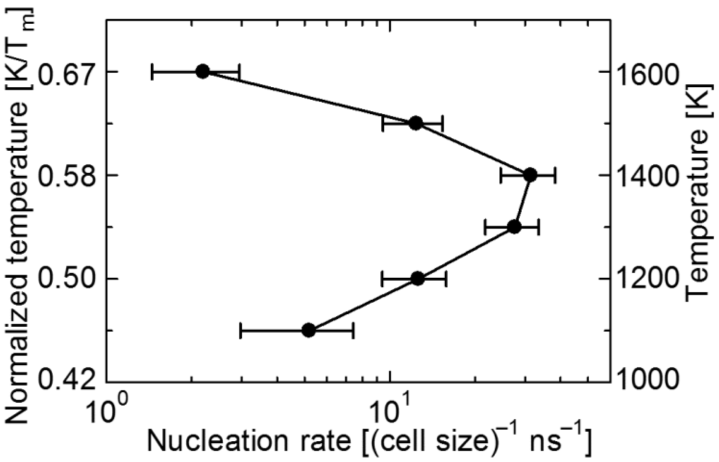

Supplementary Figure 1 | Nucleation rate as a function of temperature for homogeneous nucleation in the undercooled iron melt using Finnis-Sinclair potential [1]. The average number obtained from five replicate calculations for each temperature is plotted with error bars showing the standard deviation. A quasi-two-dimensional cell (53.4 × 53.4 × 4.3 nm^3) with 1,037,880 atoms was employed for the calculation.

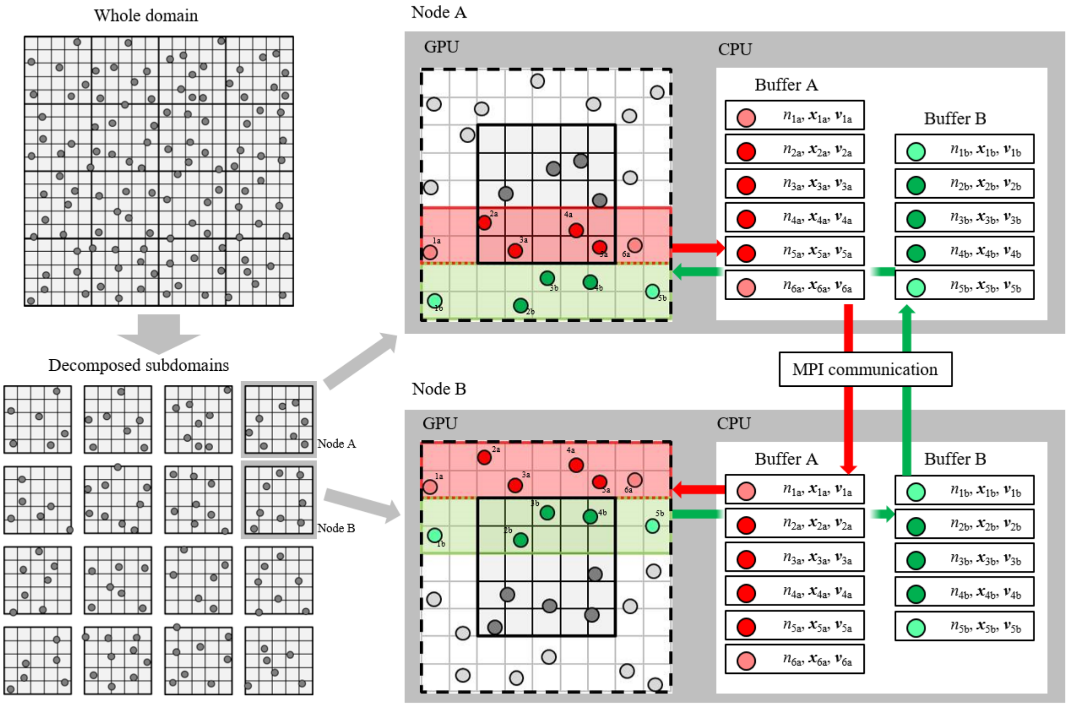

Supplementary Figure 2 | Schematic illustration of the multi-GPU parallelization. The whole simulation cell is divided into subdomains and each subdomain is assigned to one GPU. Data exchange between the GPUs is performed for the data on the subdomain boundaries via host CPUs using the Message Passing Interface (MPI).

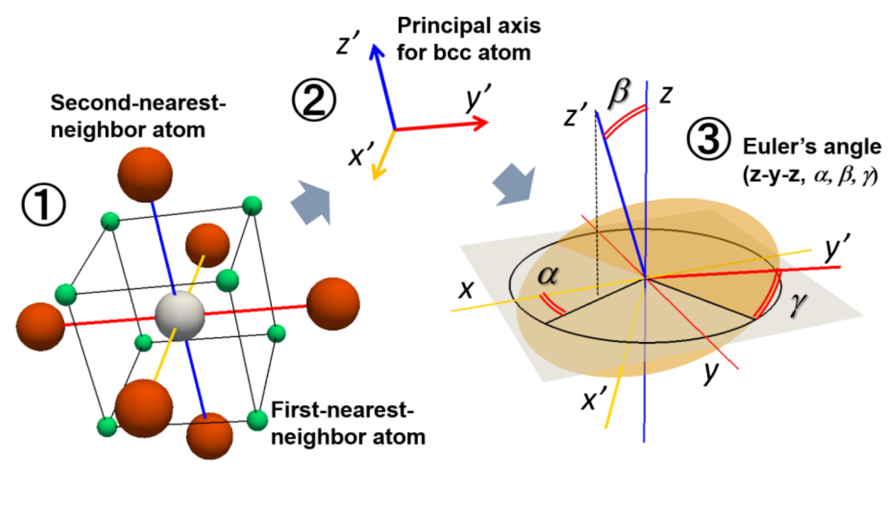

Supplementary Figure 3 | Schematic illustration of the definition of the disorientation angle of bcc atoms relative to the coordination axis.

Supplementary Note 1. Parameter set of iron for Finnis-Sinclair potential

In this study, classical molecular dynamics simulation is employed, which requires the interatomic potential. The Finnis–Sinclair (FS) potential [2] is employed for the interatomic potential between iron atoms, which is one of the most established potentials for body-centered-cubic (bcc) metals. It has been confirmed in previous studies that the FS potential can reproduce various properties of bcc materials including solidification from an undercooled iron melt [3-5]. The total energy of the FS potential, *E*, is expressed as follows:

$$E = \frac{1}{2}\sum_i \sum_j V_{ij}(r_{ij}) - A\sum_i \sqrt{\rho_i} \qquad \text{(Supplementary Equation 1)}$$

$$V_{ij}(r_{ij}) = \begin{cases} (r_{ij}-c)^2(c_0 + c_1 r_{ij} + c_2 r_{ij}^2) & (r_{ij} \le c) \\ 0 & (r_{ij} > c) \end{cases} \qquad \text{(Supplementary Equation 2)}$$

$$\rho_i = \sum_{j \ne i} \phi(r_{ij}) \qquad \text{(Supplementary Equation 3)}$$

$$\phi(r_{ij}) = \begin{cases} (r_{ij}-d)^2 + \beta \dfrac{(r_{ij}-d)^3}{d} & (r_{ij} \le d) \\ 0 & (r_{ij} > d) \end{cases}, \qquad \text{(Supplementary Equation 4)}$$

where V is the repulsive term, r_{ij} is the bond length between atoms i and j, ρ is the total electronic charge density at the site of atom i, which is constructed by the rigid superposition of atomic charge densities ϕ, A is the binding energy, c_0, c_1 and c_2 are free parameters used for fitting experimental data, c and d are cutoff parameters assumed to lie between the second-nearest- and third-nearest-neighbor atoms and β is a parameter used to introduce the maximum value of ϕ within the first-nearest-neighbor distance. The parameters for bcc iron from the original FS paper are employed [2] (Supplementary Table 1).

Supplementary Table 1. Potential parameters of iron for Finnis-Sinclair potential [2]

d [Å]	A [eV]	β	c [Å]	c_0	c_1	c_2
3.569745	1.828905	1.8	3.40	1.2371147	−0.3592185	−0.0385607

Supplementary Note 2. Disorientation angle between two grains

In general, the disorientation angle between two grains θ is defined [6,7] as

$$\theta = \cos^{-1}\left(\frac{Tr(\mathbf{R})-1}{2}\right), \qquad \text{(Supplementary Equation 5)}$$

where **R** is the rotation matrix between the two grain orientations given by

$$\mathbf{R} = \mathbf{M}_1\mathbf{M}_2^T. \qquad \text{(Supplementary Equation 6)}$$

$\mathbf{M_1}$ and $\mathbf{M_2}$ represent orientational matrices for two grains defined from the Euler angle relative to the coordination axis. The angle is minimized over the symmetry operations of the two crystals.

In this study, the disorientation angle between two grains is estimated directly from the atomistic configuration as follows (see Supplementary Figure 3). Firstly, atoms with the bcc configuration are defined by common neighbor analysis. The principal axis for the bcc atom is determined as the direction to the second-nearest-neighbor atoms, since the second-nearest-neighbor atoms of bcc atoms should be located in the orthogonal directions. Then, the Euler angle between the coordination axis and the principal axis of the bcc atom is calculated and the orientational matrix of the bcc atoms is determined as

$$\mathbf{M}_i = \mathbf{R}_z(\alpha)\mathbf{R}_y(\beta)\mathbf{R}_z(\gamma). \qquad \text{(Supplementary Equation 7)}$$

Then, the rotation matrix between two-neighboring grains is calculated as

$$\mathbf{R} = \mathbf{M}_1\mathbf{M}_2^T = \begin{pmatrix} r_{11} & r_{12} & r_{13} \\ r_{21} & r_{22} & r_{23} \\ r_{31} & r_{32} & r_{33} \end{pmatrix}. \qquad \text{(Supplementary Equation 8)}$$

Finally, the disorientation angle is obtained as

$$\theta = \cos^{-1}\left(\frac{r_{11}+r_{22}+r_{33}-1}{2}\right). \qquad \text{(Supplementary Equation 9)}$$

Supplementary Note 3. Mackenzie distribution for disorientation angle

The distribution function for a perfectly random orientation was derived by Handscomb [8] and Mackenzie [9] for materials with randomly oriented crystals. According to Mackenzie's definition [9], the distribution of the disorientation angle $p(\theta)$ consists of four ranges depending on the disorientation angle θ as follows.

$$p(\theta) = \frac{2}{15}(1-\cos\theta) \quad \left(0 \le \theta < 45^\circ\right), \qquad \text{(Supplementary Equation 10)}$$

$$p(\theta) = \frac{2}{15}\left[3\left(\sqrt{2}-1\right)\sin\theta - 2(1-\cos\theta)\right] \quad \left(45^\circ \le \theta < 60^\circ\right), \qquad \text{(Supplementary Equation 11)}$$

$$p(\theta) = \frac{2}{15}\left[\left\{3\left(\sqrt{2}-1\right)+\frac{4}{\sqrt{3}}\right\}\sin\theta - 6(1-\cos\theta)\right] \quad \left(60^\circ \le \theta < 60.72^\circ\right),$$

(Supplementary Equation 12)

$$\begin{aligned} p(\theta) = {} & \frac{2}{15}\left[\left\{3\left(\sqrt{2}-1\right)+\frac{4}{\sqrt{3}}\right\}\sin\theta - 6(1-\cos\theta)\right] \\ & - \frac{8}{5\pi}\left[2(\sqrt{2}-1)\arccos(X\cot\frac{1}{2}\theta) + \frac{1}{\sqrt{3}}\arccos(Y\cot\frac{1}{2}\theta)\right]\sin\theta \\ & + \frac{8}{5\pi}\left[2\arccos\left\{\frac{\left(\sqrt{2}+1\right)X}{\sqrt{2}}\right\} + \arccos\left\{\frac{\left(\sqrt{2}+1\right)Y}{\sqrt{2}}\right\}\right](1-\cos\theta) \quad \left(60.72^\circ \le \theta < 62.80^\circ\right) \end{aligned},$$

where

$$X = \frac{\left(\sqrt{2}-1\right)}{\left[1-\left(\sqrt{2}-1\right)^2\cot^2\frac{1}{2}\theta\right]^{\frac{1}{2}}}, \qquad \text{(Supplementary Equation 13)}$$

$$Y = \frac{\left(\sqrt{2}-1\right)^2}{\left[3-\cot^2\frac{1}{2}\theta\right]^{\frac{1}{2}}}. \qquad \text{(Supplementary Equation 14)}$$

The Mackenzie distribution defined above is shown as dashed lines in Fig. 4.

Supplementary References

1. Shibuta, Y., Oguchi, K., Takaki, T. & Ohno, M. Homogeneous nucleation and microstructure evolution in million-atom molecular dynamics simulation. *Sci. Rep.* **5,** 13534 (2015).
2. Finnis, M.W. & Sinclair, J.E. A simple empirical N-body potential for transition metals. *Philos. Mag. A* **50,** 45–55 (1984).
3. Shibuta, Y., Oguchi, K. & Suzuki, T. Large-scale molecular dynamics study on evolution of grain boundary groove of iron. *ISIJ Int.* **52,** 2205–2209 (2012).
4. Shibuta, Y., Oguchi, K. & Ohno, M. Million-atom molecular dynamics simulation on spontaneous evolution of anisotropy in solid nucleus during solidification of iron. *Scr. Mater.* **86,** 20–23 (2014).
5. Shibuta, Y., Sakane, S., Takaki, T. & Ohno, M. Submicrometer-scale molecular dynamics simulation of nucleation and solidification from undercooled melt: linkage between empirical interpretation and atomistic nature. *Acta. Mater.* **105,** 328–337 (2015).
6. Olmsted, D.L., Foiles, S.M. & Holm, E.A. Survey of computed grain boundary properties in face-centered cubic metals: I. Grain boundary energy. *Acta Mater.* **57,** 3694–3703 (2009).
7. Mason, J.K. & Schuh, C.A. The generalized Mackenzie distribution: Disorientation angle distributions for arbitrary textures. *Acta Mater.* **57,** 4186–4197 (2009).
8. Handscomb, D.C. On the random disorientation of two cubes. *Can J. Math.* **10,** 85–88 (1957).
9. Mackenzie, J.K. Second paper on statistics associated with the random disorientation of cubes. *Biometrika* **45,** 229–240 (1958).
